# Subtyping of polyposis nasi: phenotypes, endotypes and comorbidities

**DOI:** 10.1007/s40629-017-0048-5

**Published:** 2018-01-22

**Authors:** Michael Koennecke, Ludger Klimek, Joaquim Mullol, Philippe Gevaert, Barbara Wollenberg

**Affiliations:** 1 Lübeck Campus, Department of Otorhinolaryngology, University Hospital Schleswig-Holstein, Ratzeburger Allee 160, 23538 Lübeck, Germany; 2 Center for Rhinology and Allergology, Wiesbaden, Germany; 3 Rhinology Unit and Smell Clinic, Department of Otorhinolaryngology, Hospital Clinic, IDIBAPS, University of Barcelona, Barcelona, CIBERES Spain; 4 Department of Otorhinolaryngology, Ghent University, Ghent, Belgium

**Keywords:** Chronic rhinosinusitis, Nasal polyps, CRSwNP, Asthma, N‑ERD

## Abstract

**Background:**

Chronic rhinosinusitis (CRS) is a heterogeneous, multifactorial inflammatory disease of the nasal and paranasal mucosa. It has not been possible to date to develop an internationally standardized, uniform classification for this disorder. A phenotype classification according to CRS with (CRSwNP) and without polyposis (CRSsNP) is usually made. However, a large number of studies have shown that there are also different endotypes of CRS within these phenotypes, with different pathophysiologies of chronic inflammation of the nasal mucosa. This review describes the central immunological processes in nasal polyps, as well as the impact of related diseases on the inflammatory profile of nasal polyps.

**Materials and methods:**

The current knowledge on the immunological and molecular processes of CRS, in particular CRSwNP and its classification into specific endotypes, was put together by means of a structured literature search in Medline, PubMed, the national and international guideline registers, and the Cochrane Library.

**Results:**

Based on the current literature, the different immunological processes in CRS and nasal polyps were elaborated and a graphical representation in the form of an immunological network developed. In addition, different inflammatory profiles can be found in CRSwNP depending on related diseases, such as bronchial asthma, cystic fibrosis (CF), or NASID-Exacerbated Respiratory Disease (N‑ERD).

**Conclusion:**

The identification of different endotypes of CRSwNP may help to improve diagnostics and develop novel individual treatment approaches in CRSwNP.

## Introduction

Chronic rhinosinusitis (CRS) affects approximately 5–15% of the European and American population, making it a widespread health problem that creates significant costs for health systems and national economies [1, 2]. Clinically, CRS (with or without nasal polyps) in adults is defined as the presence of two or more symptoms one of which should be either nasal blockage/obstruction/congestion or nasal discharge (anterior/posterior nasal drip), ± reduction or loss of smell, ± facial pain/pressure, for more than 12 weeks [1, 3]. Secondary symptoms such as headache, fever, halitosis, cough, toothache, drowsiness, or ear pressure may also be present. More recent US and European guidelines require—in addition to two main criteria—endoscopic and/or radiological evidence of inflammatory tissue [1, 4]. Based on endoscopic examinations of the nasal cavity or imaging procedures, CRS can be differentiated into chronic rhinosinusitis with nasal polyps (CRSwNP) and chronic rhinosinusitis without nasal polyps (CRSsNP). This classification is referred to as phenotype classification.

There is currently increasing evidence to suggest that there are numerous endotypes of CRS, with different pathophysiologies and different forms of inflammation within the phenotypes CRSwNP and CRSsNP. This is particularly true for CRSwNP, which affects approximately 1–4% of the general population [1].

Histologically, nasal polyps are pale gray, edematous, sometimes also fibrous, stalked protrusions that develop in the middle nasal passage, the ethmoid bone, and the middle nasal turbinate [5]. For reasons as yet unknown, the lower nasal turbinate does not tend to form polyps [6–9]. Nasal polyps can be divided into at least four groups according to histological criteria [10–12]. At a frequency of 65–90%, edematous, eosinophilic polyps are the most common form of nasal polyps [9, 12]. Further differentiation of CRSwNP phenotypes may help to develop new therapeutic strategies that are tailored to the respective classification.

However, the partially overlapping histological features do not always permit precise classification into a certain type. Against this background, there is also a discussion about whether histological examinations could be influenced by different preoperative medications [12]. Therefore, it is currently unclear how differing therapies for the clinical treatment of nasal polyps can be determined on the basis of these classifications. For this reason, reliable and easy-to-determine biomarkers beyond classical histology would be highly desirable and could significantly improve diagnostics. However, these are not available as yet [13].

All forms of CRS appear to be caused by inflammatory changes in the sinonasal mucosa. A Th2-mediated inflammatory process is usually found in CRSwNP, whereas both Th2- and Th1-mediated processes are found in CRSsNP [1]. However, this subdivision is an over-generalization and is by no means found consistently, which is why the latest findings in this regard are described in more detail below.

## Immunology of nasal polyps

In recent years, the T cell subpopulations in chronic sinusitis and nasal polyposis have been well characterized and their biological function determined.

CD4^+^ T cells are able to differentiate into, e. g., T helper cells (Th)1, Th2, Th9, Th17, Th22, and follicular T helper (TFH) effector cells [14, 15]. The balance between these T helper subtypes is extremely important for the physiology of the mucosal immune system and can be altered by persistent inflammatory processes. Eosinophilic, Th2-dominated cell infiltration is usually seen in CRSwNP [1]. The inflammatory process is characterized by interleukin (IL)‑4‑, IL‑5‑ and IL-13-producing Th2 cells, as well as eosinophilic cationic protein (ECP) and eotaxin-1/-2/-3 [16, 17]. Each of these cyto- and chemokines has specific functions. IL-4 is a mediator and modulator of the immune and inflammatory response and is mainly produced by Th2 cells. In addition, IL-4 is able to promote the differentiation of CD4^+^ T cells into Th2 cells and at the same time inhibit interferon (IFN)-γ production and Th1 response [18, 19]. It was shown only recently that upregulation of IL-4 occurs in nasal polyps, whereas IFN-γ expression is reduced, and that IFN-γ levels do not differ significantly between nasal polyps and control tissue [17, 20, 21]. IL-5 is the most important eosinophilic activating cytokine and promotes the survival of mature eosinophils in tissue [22, 23]. IL-5 is upregulated in nasal polyps [24] and plays an important role in the pathogenesis of nasal polyps. ECP and eotaxin promote the attraction and activation of eosinophils and are also upregulated in nasal polyps [16, 17, 25].

IL-6 is a proinflammatory cytokine capable of inhibiting neutrophil recruitment [26–28] and was also found to be upregulated in CRSwNP [29, 30]. However, the differing data on regulatory molecules of the IL-6 signaling pathway do not answer the question of whether the IL-6 signaling pathway is part of the pathogenesis of CRSwNP.

Keswani and colleagues [31], as well as Cho et al. [32], found elevated expression of IL-32 in whole tissue extracts from nasal polyps. IL-32 is also described as a proinflammatory cytokine [33–38], which appears to play a role in various inflammatory disorders such as chronic obstructive pulmonary disease (COPD) and atopic dermatitis [39, 40]. Nine different isoforms of IL-32 are now known, although their functional differences remain unclear [34, 41]. Further studies are required to assess the role of IL-32 in nasal polyps in patients with chronic rhinosinusitis.

IL-25 and IL-33 are other cytokines that are produced in sinonasal epithelial cells and which promote Th2 inflammation in CRSwNP [42–44]. IL-25 is upregulated in nasal polyps and increases thymic stromal lymphopoietin (TSLP)-induced Th2 cell expansion [45, 46]. Increased expression of TSLP in the epithelium of patients with CRSwNP has already been demonstrated [47–49]. This is an IL-7-like cytokine. This effectively activates mast cells in combination with IL-1 to produce in turn Th2 cytokines, including IL-5 and IL-13 [50]. A study from Baltimore (MA, USA) showed that the sinonasal epithelial cells of patients with untreated CRSwNP show increased basal expression of IL-33 compared with sinonasal epithelial cells in patients with CRSwNP following treatment with methylprednisolone [51]. This increased expression of IL-33 in untreated polyps was confirmed by another working group [52]. IL-33 is a chemoattractant for Th2 cells and promotes the production of Th2 cytokines such as IL-4, IL-5, and IL-13. Epithelial cells of the respiratory tract are able to produce IL-33; its receptor is expressed by, e. g., eosinophils and Th2 lymphocytes [53]. IL-33 plays an important role in maintaining Th2-mediated eosinophilic inflammation [54]. Thus, polymorphisms within the IL-33 receptor gene, the interleukin-1 receptor-like 1 (IL1RL1) gene, correlate with CRS severity [55]. It is assumed that IL-25 and IL-33 are able to establish the link between epithelial cells and the Th2 response [42]; however, this needs to be investigated further.

On the other hand, the cytokines IL-25, IL-33, and TSLP have an effect on type 2 innate lymphoid cells (ILC2) [56]. These are lymphocyte-like cells that do not express any allergen-specific T cell receptors. ILCs are considered to be counterparts of Th2 cells, since both produce cytokines such as IL-5 and IL-13 [57], meaning that ILC2 activated by IL-33 and IL-25 can induce eosinophilic respiratory tract inflammation [58, 59]. ILC2 are abundant in nasal polyps and are associated with an increased number of eosinophils in the blood and tissue of patients with CRSwNP and a clinically relevant worsening of total nasal symptom score (TNSS) and asthma comorbidity [60, 61].

In addition to eotaxin-1/-2/-3, various chemokines, such as CC motif chemokine ligand 5 (CCL5 or RANTES), CXC motif chemokine ligand 8 (CXCL8 or IL-8), CCL23, CCL18, CXCL12 (stromal cell-derived factor 1 α, SDF-1 α), and CXCL13 (B cell-attracting chemokine 1, BCA-1), have been linked to the selective recruitment of inflammatory cells in the mucosa in CRSwNP. RANTES was one of the first identified chemokines found to be upregulated in nasal polyps [62, 63]. RANTES is a member of the CC chemokine family and a strong chemoattractant for eosinophils and T‑lymphocytes, but not for neutrophils, and is primarily secreted by nasal epithelial cells [62, 64, 65]. RANTES is also expressed and secreted in nasal polyps. Interestingly, nasal polyps with a high number of eosinophils exhibit significantly increased RANTES gene and protein expression. Consequently, increased RANTES expression results in an increased number of eosinophils in tissue. Therefore, RANTES likely also plays an important role in the mobilization of eosinophils in nasal polyps.

CXCL8 (IL-8), which attracts neutrophils and eosinophils into the nasal mucosa, provided these have been previously activated by IL-5, plays a further role in nasal polyp inflammation [66]. IL-8, however, is considered more of an unspecific marker for CRSwNP. Although altered levels of IL-8 were identified in nasal polyps [30, 66–69], upregulation does not correlate significantly with the formation of nasal polyps [66].

Poposki and colleagues [70] were able to show strong production of CCL23 in nasal polyps, which was largely colocalized with ECP, indicating predominantly eosinophilic CCL23 production in nasal polyps. CCL23 is a chemoattractant for monocytes, dendritic cells, and lymphocytes, and it has been shown that CCL23 induces endothelial cell migration via the CC motif chemokine receptor CCR1, which is also upregulated in nasal polyps [70–73]. It has been shown for Th2 cytokines such as IL-4 and IL-13 that these induce CCL23 expression in monocytes [74].

In addition, significantly increased CCL18-mRNA expression was found in nasal polyps and the inferior tubinates [75]. M2 macrophages and mast cells were identified in CRSwNP that express CCL18, which can be induced by the Th2 cytokines IL4-, IL-13, and IL-10 [75]. Since the related receptor CCR8 was only recently identified, the role of CCL18 in the pathogenesis of CRSwNP has not yet been investigated in detail [76]. However, this discovery will help clarify the role of CLL18 in CRSwNP.

Both B cells and the antibody fractions IgA and IgE were found to be elevated in patients with CRSwNP [77, 78]. B cells express IgA, which triggers the degranulation of eosinophils and represents a possible link to CRSwNP [77]. In this context, it was shown that the chemokines CXCL12 (SDF-1 α) and CXCL13 (BCA-1) are present at elevated levels in nasal polyps. Both attract B cells. Furthermore, the receptors for SDF-1 α (CXCR4 and CXCR7) and BCA-1 (CXCR5) are also present at elevated levels [79]. Consequently, the expression of SDF-1 α and BCA-1 in nasal polyps could be important for the recruitment and maintenance of B cells. In addition, the increased IgA levels imply an important role for B cells in the pathogenesis of CRSwNP.

**Fig. 1:**
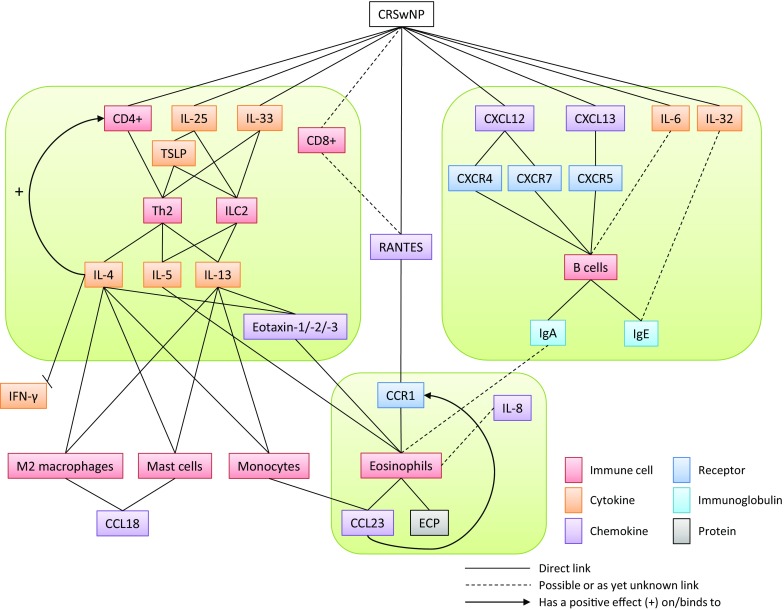
The immunological processes in nasal polyps. CRSwNP can be divided into three different endotypes: the T cell‑/Th2 cytokine-based endotype, the B cell‑/IgE-based endotype, and the eosinophil-based endotype. This gives rise to different treatment approaches in CRSwNP. *CRSwNP* chronic rhinosinusitis with nasal polyps; *CD* cluster of differentiation; *IL* interleukin; *TSLP* thymic stromal lymphopoietin; *Th* T-helper; *ILC* innate lymphoid cell; *CXCL* CXC motif chemokine ligand; *CXCR* CXC motif chemokine receptor; *Ig* immunoglobulin; *IFN* interferon; *CCR* CC motif chemokine receptor; *CCL* CC motif chemokine ligand

Immunological processes are complex and involve a multitude of cell types and proteins. Fig. 1 shows the immunological processes known in nasal polyps.

## Phenotypes of CRS

For some time now, CRS has been divided into different entities. The likely simplest classification into CRSsNP and CRSwNP is based on endoscopic or rhinoscopic findings.

## Comorbidities of CRS

Another traditional classification of CRS is based on its association with other diseases, e. g., the comorbidity of CRSwNP with cystic fibrosis (CF), aspirin intolerance syndrome (AIS) or NASID-Exacerbated Respiratory Disease (N‑ERD), inhalation allergies, immunodeficiency syndromes, allergic fungal sinusitis, Wegener’s granulomatosis, and bronchial asthma [1].

With regard to CF, nasal polyps are present in approximately 40% of these patients [80]. These polyps show a more neutrophilic inflammation pattern with overexpression of IL-1 β, IL-8, IL-21, myeloperoxidase (MPO), and IL-17-producing Th17 cells [17, 20, 81]. As part of this, IL-1 β and IL-17 mediate T cell differentiation towards a Th17 phenotype [82]. CF is caused by a defect in the transport of chloride and bicarbonate (cystic fibrosis transmembrane conductance regulator protein, CFTR) [83, 84]. To diagnose CF, chloride sweat tests (the sweat of CF patients contains more than 60 mmol/l chloride, or in the case of newborns, over 90 mmol/l chloride) and molecular genetic screening methods, among others, are performed [83, 84].

The prevalence of N‑ERD among patients with CRSwNP is approximately 16%, while, conversely, up to 96% of N‑ERD patients have nasal polyposis [1, 9]; therefore, a direct pathophysiological link between N‑ERD and nasal polyps has been discussed for some time. The cytokine profile of nasal polyps in N‑ERD patients shows the typical components of a Th2 disease, as well as eosinophilia and significant upregulation of IL-4 and IL-5 [85]. In addition, significantly increased CCL23 expression is seen in nasal polyps in patients with N‑ERD [70]. However, the most striking feature is the increased IFN-γ-expression of eosinophils [85], which is typically associated with Th1 inflammation. To verify the suspected diagnosis of N‑ERD, oral provocation testing is considered the gold standard alongside bronchial and nasal provocation tests [86–88]. Research is underway into the development of reliable in vitro laboratory techniques as a diagnostic alternative to provocation testing [87–89]. All laboratory chemical tests to date have been based on detecting the impaired arachidonic acid metabolism in this disease [87–90].

The influence of inhalation allergies on the pathogenesis of nasal polyps is the subject of controversy [1]. In particular, the high prevalence of allergic diseases in the European population makes direct correlations challenging [91]. A study by Perić and colleagues in 2012 [92] investigated the influence of allergies in patients with nasal polyps. This study involving 30 patients—13 with allergies, 17 without allergies—investigated the cytokine and chemokine levels in nasal secretions. The concentrations of IL-4, IL-5, IL-6, and tumor necrosis factor (TNF)-β were significantly higher in allergic patients compared with non-allergic patients. In addition, the number of eosinophils in nasal polyps was significantly higher in allergic patients compared with the non-allergic group. Moreover, it has been shown that both IL-6 and the Th2 cytokines IL-4 and IL-5 are more strongly expressed in patients with allergic rhinitis [93, 94]. Interestingly, IL-6 was more highly expressed in nasal polyps in allergic individuals than in nasal polyps in non-allergic individuals [92, 95], suggesting that IL-6 and the IL-6 signaling pathway could play a potential role in CRSwNP in patients with inhalation allergies. Furthermore, studies on the pathogenesis of nasal polyps have not demonstrated a link to elevated TNF-β as yet. Also known as lymphotoxin (LT), TNF-β is produced by Th1 cells, in addition to IFN-γ and TNF-α, and promotes cellular inflammation [96]. As such, TNF-β may be a potential marker for allergic nasal polyps alongside IL-6.

Immunodeficiencies on the humoral and cellular levels have also been associated with CRSsNP and CRSwNP [97–100]. Defects in the production or function of IgA and IgG immunoglobulins, cellular effects at the B cell level, T cells, neutrophils/monocytes, and complement defects play an important role here [97–100].

The prevalence of allergic fungal rhinosinusitis (AFRS) or eosinophilic fungal rhinosinusitis (EFRS) in patients with nasal polyposis is about 9–12% [101, 102] and shows Th2-mediated inflammation with varying levels of Th2 cytokines [103]. AFRS-/EFRS-related nasal polyps exhibit a significantly higher level of IgE and IL-5. However, further investigations are needed to assess the role of AFRS/EFRS in the pathogenesis of CRSwNP, since the so-called “fungal hypothesis” as the cause of CRSwNP can be considered disproved [1].

Furthermore, specific forms of CRS have been described in sarcoidosis, Wegener’s granulomatosis, congenital or acquired disorders of mucociliary clearance and/or ciliary activity, as well as Churg–Strauss syndrome [1, 100, 104].

Bronchial asthma is particularly frequently associated with CRSwNP: approximately 40% of patients with CRSwNP also suffer from bronchial asthma [9]; conversely, around 7% of asthma patients have nasal polyps [1]. However, the correlation between asthma and CRSwNP is poorly understood as yet. In a 2013 study, nasal secretions from 40 patients with nasal polyps (20 with and 20 without asthma) were investigated for cytokine expression [105]. IL-5, IL-6, and IL-10 levels were significantly increased in asthmatic patients with nasal polyps compared with non-asthmatic patients with nasal polyps. However, it is not possible to say with any certainty at present that this alteration can be attributed to the correlation with asthma. Another study by Nabavi and colleagues [106] found no differences in the serum expression of IL-4, IL-13, and IFN-γ between asthmatic patients with CRSwNP and non-asthmatic patients with CRSwNP. However, increased IgE levels were found. To date, IL-5 and IgE were identified as indicators for patients with CRSwNP and additional asthma [107]. Only recently, Tomassen and colleagues were able to show that there is a correlation between clinical characteristics and inflammatory endotypes of CRS. Results showed that patients with high IL-5 levels had the highest prevalence of nasal polyps and asthma. In contrast, patients with low IL-5 levels were primarily classified as CRSsNP. Thus, the prevalence of asthma increases significantly depending on this endotype classification in patients with CRSwNP. IL-5 remains the main factor determining the phenotype with nasal polyps and asthma, thereby gaining in importance as a therapeutic target [30].

## Endotypes of CRS

Four distinct endotypes of CRSwNP, albeit it with clearly overlapping characteristics, have been defined in a classification scheme [108] in a current review article on endotype-based subtyping of CRSwNP:

- T cell‑/Th2 cytokine-based endotype
- Eosinophil-based endotype
- B cell‑/IgE-based endotype
- Cysteinyl-based endotype

## Endotypes of CRS with various comorbidities

**Fig. 2:**
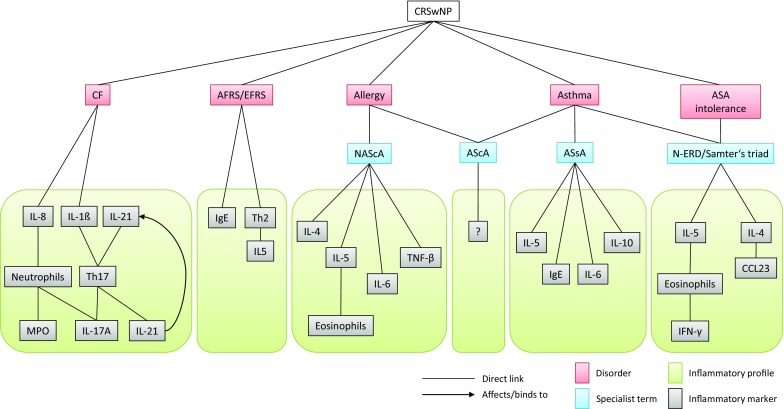
The affect of various comorbidities of CRSwNP on the inflammatory profile in nasal polyps: different comorbidities of CRSwNP affect the development of endotypes of nasal polyps. As a result, the classic inflammatory profile in nasal polyps shifts and gains additional specific markers. When associated with cystic fibrosis, the inflammatory profile of nasal polyps changes extensively. *CRSwNP* chronic rhinosinusitis with nasal polyps; *CF* cystic fibrosis; *AFRS* allergic fungal rhinosinusitis; *EFRS* eosinophilic fungal rhinosinusitis; *NAScA* non-asthmatic sinusitis with allergy; *AScA* asthmatic sinusitis with allergy; *ASsA* asthmatic sinusitis without allergy; *N‑ERD* NASID-Exacerbated Respiratory Disease; *IL* interleukin; *Th* T-helper; *MPO* myeloperoxidase; *Ig* immunoglobulin; *TNF* tumor necrosis factor; *CCL* CC motif chemokine ligand

The various comorbidities of CRSwNP affect endotype expression and the associated inflammatory profile of nasal polyps (Fig. 2). Therefore, the diagnosis of comorbidities should be included in endotype classification. This requirement has not been taken into consideration in previous publications on endotype classification in CRSwNP and could represent an important step on the way to personalized medicine or precision medicine in CRSwNP.

## Novel treatment approaches based on endotype classification

Endotype classification gives rise to a number of novel treatment approaches in CRSwNP, e. g., the use of biologics such as dupilumab (anti-IL-4/13), mepolizumab (anti-IL-5), or omalizumab (anti-IgE) [109, 110].

These newly emerging therapies are aimed at a specific pathophysiological signaling pathway. However, careful selection of the patient population is required to successfully implement such endotype-based treatment approaches. Positive treatment outcomes have already been demonstrated in CRSwNP with the above-mentioned monoclonal antibodies against IgE, IL-5, and IL-4/13 [111].

To date, however, only a selected patient collective has benefited from these developments. More precise endotype classification of patients could potentially help to optimize treatments. A discussion will be required, for example, as to whether treatment directed against a specific endotype is sufficient for certain patient populations or whether combination therapy might be necessary.

With regard to possible applications in the routine treatment of CRS patients, it is essential to mention here that there are no approved preparations for the indications CRS and CRSwNP. Therefore, treatment performed outside clinical trials has usually been carried out in patients receiving these substances for other indications or as individual treatment attempts in the form of off-label therapy.

## Conclusion

CRS is a heterogeneous group of inflammatory disorders of the nasal and paranasal sinus mucosa. In clinical routine, the disorder continues to be subdivided into CRSsNP and CRSwNP on the basis of phenological characteristics.

However, there is no doubt that there are multifactorial pathophysiologies for CRSwNP in particular. These different endotypes have different signaling pathways, from the process of initiation of inflammation, its maintenance, and its chronification to tissue alteration. In addition to the “classic” CRSwNP endotype of Th2-based and eosinophil-dominated inflammation, there are other endotypes that suggest different therapeutic approaches [111].

Hitherto, endotype classifications have neglected the fact that disorders associated with CRSwNP may point to the endotype and the typical inflammatory pattern that ensues. These should therefore be included in new classifications to be developed in the future.

Against this background, topical nasal glucocorticosteroids will remain the basic treatment in CRS and surgical approaches will continue to be indispensable. However, the addition of endotype-based individualized treatments with potential biologicals to these “basic treatment options” could help to put into effect the principle of “personalized medicine” in CRS in the future.

## Abbreviations

AFRS: Allergic fungal rhinosinusitis
AIS: Aspirin-intolerance syndrome
AFS: Allergic fungal sinusitis
AScA: Asthmatic sinusitis with allergy
ASsA: Asthmatic sinusitis without allergy
BCA: B cell-attracting chemokine
CD: Cluster of differentiation
CF: Cystic fibrosis
CFTR: Cystic fibrosis transmembrane conductance regulator protein
CRS: Chronic rhinosinusitis
CRSsNP: Chronic rhinosinusitis without nasal polyps
CRSwNP: Chronic rhinosinusitis with nasal polyps
CCL: CC motif chemokine ligand
CCR: CC motif chemokine receptor
CXCL: CXC motif chemokine ligand
CXCR: CXC motif chemokine receptor
ECP: Eosinophilic cationic protein
EFRS: Eosinophilic fungal rhinosinusitis
IgA: Immunoglobulin A
IgE: Immunoglobulin E
IFN: Interferon
IL: Interleukin
ILC: Innate lymphoid cells
MPO: Myeloperoxidase
NAScA: Non-asthmatic sinusitis with allergy
N‑ERD: NASID-exacerbated respiratory disease
SDF: Stromal cell-derived factor
TFH: Follicular helper T cells
Th: T helper
TNF: Tumor necrosis factor
TNSS: Total nasal symptom score
TSLP: Thymic stromal lymphopoietin
